# Mpox Knowledge Graph: a comprehensive representation embedding chemical entities and associated biology of Mpox

**DOI:** 10.1093/bioadv/vbad045

**Published:** 2023-04-03

**Authors:** Reagon Karki, Yojana Gadiya, Andrea Zaliani, Philip Gribbon

**Affiliations:** Discovery Research ScreeningPort, Fraunhofer Institute for Translational Medicine and Pharmacology (ITMP), Schnackenburgallee 114, 22525 Hamburg, Germany; Fraunhofer Cluster of Excellence for Immune-Mediated Diseases (CIMD), Theodor Stern Kai 7, 60590 Frankfurt, Germany; Discovery Research ScreeningPort, Fraunhofer Institute for Translational Medicine and Pharmacology (ITMP), Schnackenburgallee 114, 22525 Hamburg, Germany; Fraunhofer Cluster of Excellence for Immune-Mediated Diseases (CIMD), Theodor Stern Kai 7, 60590 Frankfurt, Germany; Discovery Research ScreeningPort, Fraunhofer Institute for Translational Medicine and Pharmacology (ITMP), Schnackenburgallee 114, 22525 Hamburg, Germany; Fraunhofer Cluster of Excellence for Immune-Mediated Diseases (CIMD), Theodor Stern Kai 7, 60590 Frankfurt, Germany; Discovery Research ScreeningPort, Fraunhofer Institute for Translational Medicine and Pharmacology (ITMP), Schnackenburgallee 114, 22525 Hamburg, Germany; Fraunhofer Cluster of Excellence for Immune-Mediated Diseases (CIMD), Theodor Stern Kai 7, 60590 Frankfurt, Germany

## Abstract

**Summary:**

The outbreak of Mpox virus (MPXV) infection in May 2022 is declared a global health emergency by WHO. A total of 84 330 cases have been confirmed as of 5 January 2023 and the numbers are on the rise. The MPXV pathophysiology and its underlying mechanisms are unfortunately not yet understood. Likewise, the knowledge of biochemicals and drugs used against MPXV and their downstream effects is sparse. In this work, using Knowledge Graph (KG) representations we have depicted chemical and biological aspects of MPXV. To achieve this, we have collected and rationally assembled several biological study results, assays, drug candidates and pre-clinical evidence to form a dynamic and comprehensive network. The KG is compliant with FAIR annotations allowing seamless transformation and integration to/with other formats and infrastructures.

**Availability and implementation:**

The programmatic scripts for Mpox KG are publicly available at https://github.com/Fraunhofer-ITMP/mpox-kg. It is hosted publicly at https://doi.org/10.18119/N9SG7D.

**Supplementary information:**

Supplementary data are available at *Bioinformatics Advances* online.

## 1 Introduction

The recent coronavirus disease 2019 (COVID-19) pandemic has drastically changed the way research and scientific studies operate in areas of infectious and epidemic diseases. Although new discoveries and uncovering pathophysiology are the ultimate expectations, a new aspect that has been crucial to these is the response time (Khanna *et al.*, 2020). Despite the expertise and technologies of the highest levels in hospitals, pharmaceutical companies and research institutes, the response was not always timely. This clearly was the impact of lack of preparedness and since then we are determined to avoid experiencing the same chaos in future epidemics (Villa *et al.*, 2020). One of the setbacks in such a situation was the unavailability of enough research data with metadata compliant with Findable, Accessible, Interoperable and Reproducible (FAIR) data principles, consequently leading to mapping gaps between different domains of scientific studies. A number of efforts have emerged since then to harmonize sparse data and better understand the etiology of the disease (Harrison *et al.*, 2021; Schmidt *et al.*, 2021).

The ongoing multi-country outbreak of Mpox virus (MPXV) which started in May 2022 (https://www.ecdc.europa.eu/en/Mpox-outbreak) has been declared a global health emergency and stands as another potential threat of pandemic. Unfortunately, the etiology of MPXV is not known and therefore, there is an urgent need to decipher it. This involves identifying the involvement of viral and host proteins in the infection, their interactions, virus replication biology and potential drug candidates to perturb viral processes and mechanisms within the host. Additionally, from drug discovery and therapeutic perspective, it is important to know active molecules and their pharmacology either as a direct effect on viral–host interactions or as cellular toxicology effects. Understanding all the above-mentioned aspects will help accelerate drug repurposing and drug discovery processes. In this work, we have created a comprehensive Mpox Knowledge Graph (KG) that represents chemical-specific information such as chemicals and drugs active against MPXV along with their side effects, biological-specific information such as proteins, and their associated biological processes. The KG is represented with standard ontologies aligning it with the FAIR data principles. Furthermore, the KG is available in various graph formats to facilitate data handling and processing as required by the scientific community.

## 2 Materials and methods

**Fig. 1.**
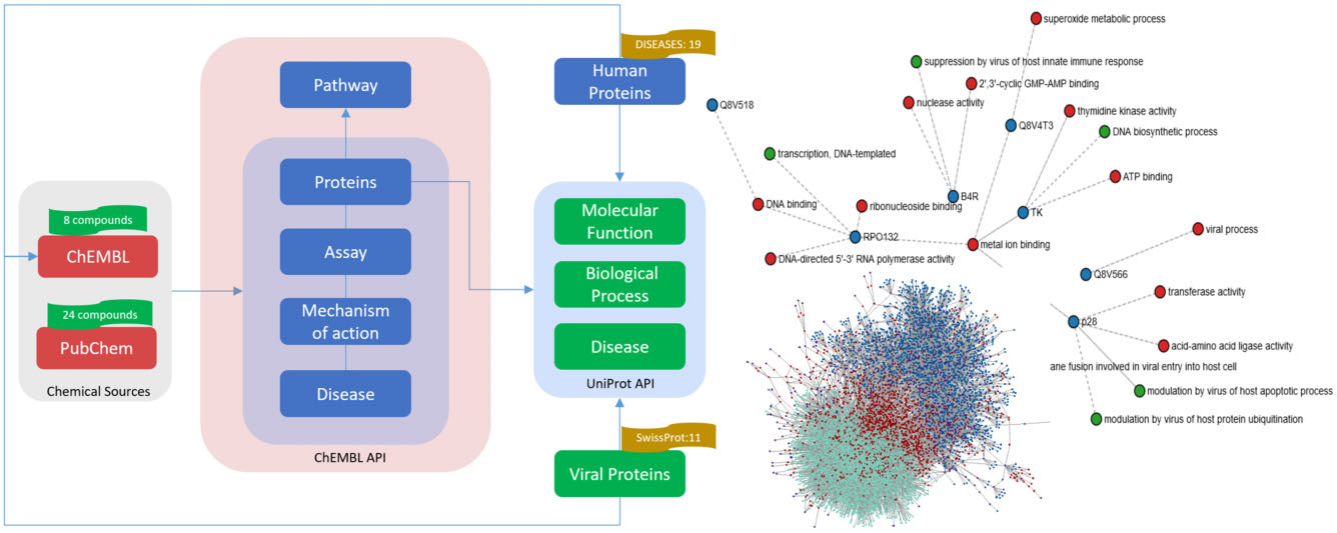
A schematic representation of the KG workflow (left) and visualization of the KG (right)

The overall methodology used for the creation of the Mpox KG is divided into three main steps (i) biological/chemical resources identification, (ii) data harmonization and standardization and (iii) KG generation (Fig. 1).

### 2.1 Resource identification

The chemical compounds used or tested against MPXV were retrieved from public chemical data resources, i.e. PubChem (Kim *et al.*, 2021), ChEMBL (Davies *et al.*, 2015) databases (last accessed: 12 January 2022). We queried PubChem with NCBI Taxonomy Identifier (ID) of the virus (NCBITaxon: 10244) and the associated chemicals were listed under the table ‘Chemicals and Bioactivities’ and sub-table ‘Tested Compounds’. Since ChEMBL has its independent ontology for taxonomy, the database was queried using ChEMBL ID for MPXV (i.e. CHEMBL613120). Afterwards, we selected chemicals with pChEMBL value > 6 from either binding or functional assays since this condition ensures bioactivity of a given chemical. In general, this value is half-maximal response concentration/potency/affinity on a negative logarithmic scale. Next, using the taxonomy ID of MPXV, we collected reviewed protein entries (Swiss-Prot) from UniProt (Apweiler *et al.*, 2004). For human proteins, we queried DISEASES, a human disease database, with DOID: 3292 (Grissa *et al.*, 2022). Lastly, Open Targets Platform was used to fetch information about the ‘druggability’ of proteins reported from studies (Ochoa *et al.*, 2021).

### 2.2 Programmatic methods for data fetching and harmonization

The programmatic scripts and methods written were written in python (version 3.10) and are available at https://github.com/Fraunhofer-ITMP/mpox-kg. Firstly, we converted PubChem IDs to ChEMBL IDs because the information about chemical-associated proteins, assays, mechanism of actions, pathways and diseases can be fetched from the ChEMBL API using ChEMBL IDs. After combining the identified proteins with proteins from UniProt and DISEASES, we used the UniProt API to extend the information on molecular functions, biological processes, sequences, pathways and diseases. Furthermore, we identified additional ChEMBL compounds that target proteins collected in the workflow and repeated the aforementioned steps of using ChEMBL and UniProt API. Lastly, we mapped and harmonized chemical and protein names as they were collected from different resources and had different identifiers. For example, PubChem entry with compound ID 16124688 is registered in ChEMBL as CHEMBL1257073. We have used ChEMBL IDs for standard and uniform representation of chemicals. Similarly, UniProt IDs were converted to HUGO names as it helps researchers to readily identify a given protein.

### 2.3 Construction of Mpox KG

The Mpox KG is represented in the form of semantic triples using Biological Expression Language (BEL) with metadata annotation on nodes and relations using the PyBEL framework (Hoyt *et al.*, 2018). PyBEL is a software tool built to facilitate data parsing, semantics validation and visualization of data generated in BEL format. The framework provides a library of functions for exploring, querying and analyzing the KG. Moreover, the KG is exported to other formats such as json, csv, sql, graphml and Neo4j which enables systematic comparison or integration with other KGs. The KG is hosted publicly in NDExbio platform under the URL: https://doi.org/10.18119/N9SG7D (Pillich *et al.*, 2021).

## 3 Results

While literature and public data resources contain sparse MPXV-related information, our approach has reached out to various resources and built a bridge between the chemical and biological worlds, thus yielding a comprehensive KG. Starting from chemicals associated with MPXV, we were able to identify corresponding assays with bioactivity, target proteins and their biological processes. Moreover, with MPXV and human proteins, we not only summoned knowledge about the aforementioned aspects but also identified chemicals targeting these proteins.

Our query from PubChem retrieved 24 chemicals, all of which were successfully mapped to corresponding ChEMBL IDs. Similarly, eight chemicals were retrieved from ChEMBL, out of which six were identical with the PubChem chemicals. The search in UniProt fetched 11 MPXV proteins whereas DISEASES returned 19 human proteins. Using these results as the primer for the KG, we created a KG using ChEMBL and UniProt API. The KG comprised 9117 nodes and 44 516 relationships where we have identified 565 putative drugs targeting human and viral proteins. The full summary of KG statistics is available in Supplementary Figures S1 and S2. The proteins represented in the KG were further labeled with ‘druggability’ information using Open Targets (Supplementary Table S1). Additionally, we performed a sequence similarity search for the MPXV proteins and identified human homologs with sequence identity >35%. These results are provided in Supplementary Table S2.

**Fig. 2.**
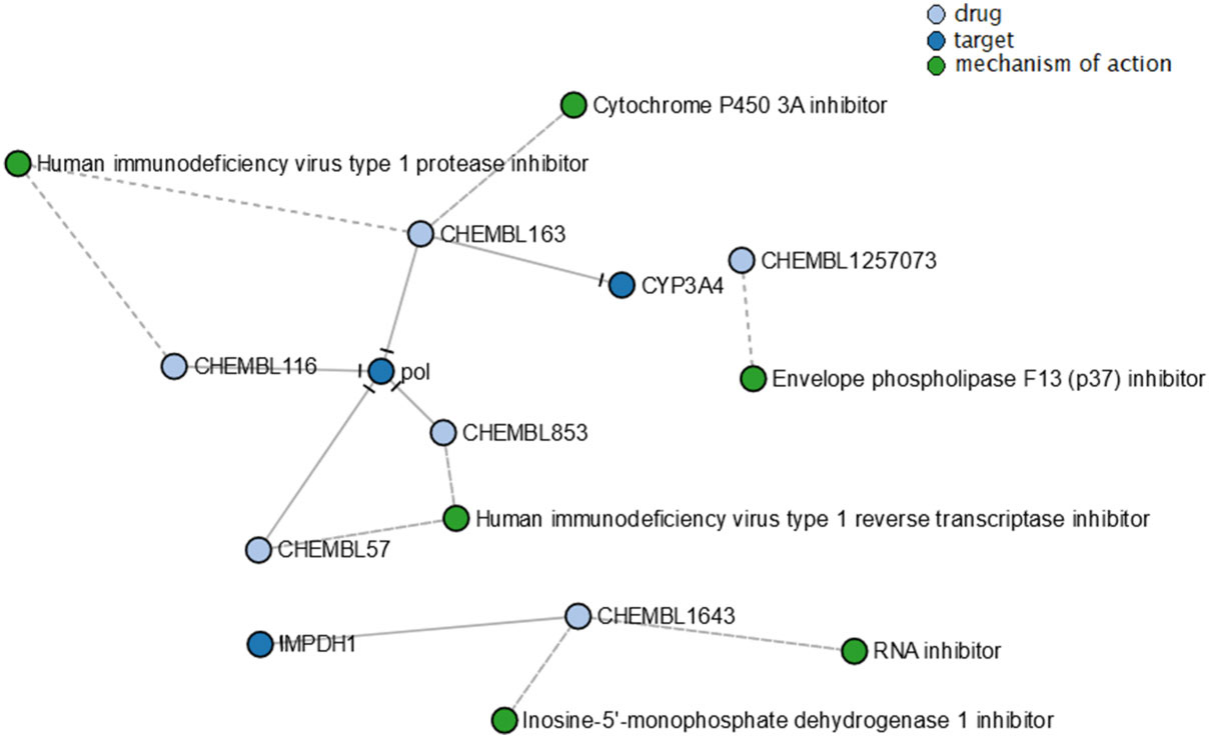
A section of the KG showing drugs, their target(s) and mechanism of action(s)

As there are no Mpox-specific drugs, we tried to identify drugs that were used against similar viruses. Our query to the KG identified 15 drugs that were in different phases of clinical trials. After filtering out for drugs that were in Phase IV, 12 drugs remained (Supplementary Table S3). To this, we filtered the list by retaining drugs with known mechanism of action(s), which resulted in 6 drugs (Fig. 2). Out of these, Ribavirin (CHEMBL1643) has been known to be a direct inhibitor of human IMPDH1, a protein involved in the regulation of cell growth (Te *et al.*, 2007). Likewise, Nevirapine (CHEMBL57) and Zalcitabine (CHEMBL853) have been shown to function as human immunodeficiency virus (HIV) type 1 reverse transcriptase inhibitors (pol) by inhibiting the corresponding protein (de Clercq, 2005; Ghosn *et al.*, 2009). Similarly, Amprenavir (CHEMBL116) and Ritonavir (CHEMBL163) are other known pol inhibitors, with the latter also functioning as human CYP3A4 inhibitor (Sadler *et al.*, 1999; Sevrioukova and Poulos, 2010).

In order to decipher any possible link between Mpox proteins and pol, we performed a BLAST analysis against Mpox proteins (Target database: UniProtKB Swiss-Prot and Mpox Taxonomy: 10244) to find their relatedness and discover the potential to repurpose its drugs against Mpox. The search identified 6 proteins with overlapping amino acids, all in the C-terminal of the protein. Among these, OPG148 (UniProt: A0A7H0DNC0) had the best overlap with 23 amino acids mapped across 97 amino acids of both proteins (Supplementary Fig. S4). Since, Mpox does not have any reported protein–ligand interaction so far, we extended the BLAST search with OPG148 [Target database: UniProtKB with 3D structure (PDB)] to identify Mpox-related proteins with ligand bound conformations. The analysis identified orthologous OPG148 (UniProt: P20995) in Vaccinia virus with 97.2% similarity (Supplementary Fig. S5). Afterwards, we explored its PDB structures and filtered for identifiers with ligands bound to the ortholog. We found that PDB structures 4YGM and 4YIG had Uracil (CHEMBL566) bound to the ortholog (Burley *et al.*, 2023). Next, we super-imposed Uracil onto Nevirapine and Zalcitabine to find out its structural resemblance. We found that Uracil-Zalcitabine had a complete substructure match while Nevirapine, although partial, had a good 3D super-imposition (Supplementary Fig. S6). Through this approach, we could swiftly identify candidate ligands for Mpox OPG148 using its ligand-complexed ortholog protein.

Lastly, we identified Tecovirimat (CHEMBL1257073) in the KG which has been previously reported to function as Envelope phospholipase F13 (p37) inhibitor (Yang *et al.*, 2005). Tecovirimat is one of the smallpox-specific drugs tested against Mpox and has been proven to induce anti-viral effects in Orthopoxvirus infections (Duraffour *et al.*, 2015). With these lines of evidence, we have short-listed FDA-approved drugs which bear the potential to be repurposed against Mpox and therefore suggest the need of further research and investigation.

## 4 Discussion

The COVID-19 pandemic has alerted the scientific community at different levels such as identifying early predictors, understanding pathogen biology and consequent pathophysiology, selecting first line of treatments, identifying putative drugs, and enabling data availability which is crucial for all sorts of research activities (Bibi *et al.*, 2022; Domingo-Fernández *et al.*, 2021). The lesson learned from COVID-19 is to be prepared and act immediately to avoid the next unprepared ‘COVID-19-esque’ situation. In this regard, aligning with FAIR data principles, we have created a Mpox KG which is a comprehensive representation of biological and chemical entities associated with MPXV. Our analysis identified six drugs with known mechanism of actions and their target proteins which could be useful in upcoming Mpox studies. This highlights a straightforward application of the KG as it allows identification and selection of putative active Mpox-related chemicals. To our knowledge, ours is the first KG in MPXV research.

One significant strength of our KG is that it not only embeds, harmonizes and visualizes entities but also serves as a primer for downstream analyses. For example, a chemoinformatician can readily run similarity search analyses using the chemicals represented in the KG. Similarly, a biologist working with a certain protein and chemical can quickly find out other chemicals targeting the same protein. We know that this approach is not without limitations but embedding KGs in drug-discovery process allows faster and innovative hypotheses generation. Considering these, we aim to facilitate ongoing and upcoming MPXV studies by serving a useful resource to different research groups and therefore will continue to update the KG actively. One of our next updates will include annotation of proteins with MPXV-specific omics data. Moreover, we plan to explore functional interactions of orthologous proteins in other Orthopoxviruses. Finally, we plan to reach out to other public resources for enriching the knowledge in the KG.

## Authors’ contributions

R.K. ideated and implemented the KG. R.K. Y.G., A.Z. and P.G. wrote the article.

## Supplementary Material

vbad045_Supplementary_Data
