# Supplementary material for: Mpox Knowledge Graph: a comprehensive representation embedding chemical entities and associated biology of Mpox
Source: Bioinform Adv. 2023 Apr 3;3(1):vbad045. doi: 10.1093/bioadv/vbad045 (PMC10181838; doi:10.1093/bioadv/vbad045)
Supplement: vbad045_Supplementary_Data [file vbad045_supplementary_data.pdf]

Supplementary File

Outline

1. Mpox Knowledge Graph in numbers
2. Proteins from 'druggability' family
3. Viral-host protein predictions
4. Clinical Trial (Phase IV) drugs in Virus Diseases
5. BLAST search for HIV reverse transcriptase (pol) and Mpox Protein OPG148 (UniProt: A0A7H0DNC0)
6. Structural Similarity between Uracil- Nevirapine and Uracil- Zalcitabine

Supplementary Text

1. Knowledge Graph in numbers

The Mpox KG (**Supplementary Figure 1)** is composed of 9117 nodes out of which 4220, 2356, 2181 and 360 nodes account for pathology, biological process, abundance and protein respectively. The pathology is further sub-divided to namespaces SideEffect and Disease where the corresponding numbers are 3315 and 905. Likewise, biological process is sub-divided to namespaces Gene Ontology Biological Process (GOBP), Gene Ontology Molecular Function (GOMF), Reactome and Mechanism of Action (MOA) and the corresponding numbers are 846, 568, 810 and 132. The abundance consisted of ChEMBL assays and chemicals where the numbers are 1732 and 449 respectively. Lastly, there are 10 viral proteins and the rest 360 are human proteins (**Supplementary Figure 2**).

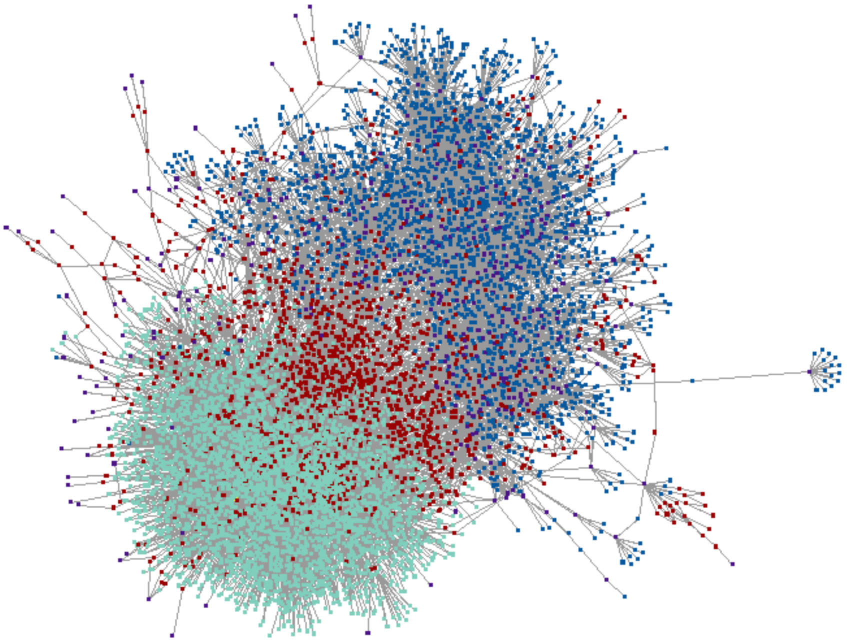

Supplementary Figure 1: A snapshot of the Mpox KG

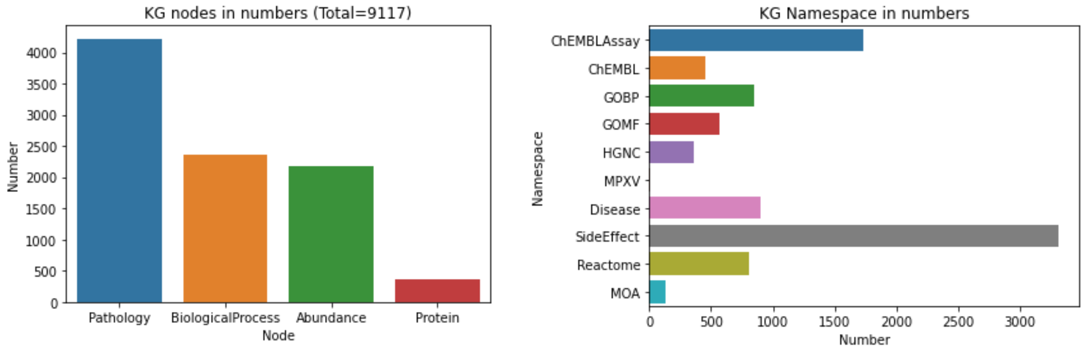

Supplementary Figure 2: Distribution of different nodes (left) and namespaces (right) in KG

2. Proteins from "druggability" family

Using Open Targets, we have annotated the proteins in KG with the information whether they belong to the druggable family of proteins. We found out that 255 proteins out of 377 belonged to the druggable family of proteins (**Supplementary Table 1**).

Protein	Druggable Family	Protein	Druggable Family	Protein	Druggable Family	Protein	Druggable Family
ABCB1	Yes	CHRNB4	Yes	HDAC2	Yes	NR3C2	Yes
ABCC8	Yes	CNR1	Yes	HDAC3	Yes	OPRL1	Yes
ACE	Yes	CNR2	Yes	HDAC8	Yes	OR10G6	No
ACHE	Yes	COMT	Yes	HIF1A	Yes	OR51E2	Yes
ADORA1	Yes	CSNK2A1	Yes	HPGD	Yes	P2RX1	Yes
ADORA2B	Yes	CSNK2B	No	HRAS	Yes	P2RX3	Yes
ADORA3	Yes	CYB5R3	No	HRH1	Yes	P2RX4	Yes
ADRA1A	Yes	CYP11B1	Yes	HRH2	Yes	P2RY2	Yes
ADRA1B	Yes	CYP11B2	Yes	HRH3	Yes	PDE3A	Yes
ADRA1D	Yes	CYP17A1	Yes	HRH4	Yes	PDE4A	Yes
ADRA2A	Yes	CYP19A1	Yes	HSD11B1	Yes	PDE4B	Yes
ADRA2B	Yes	CYP1A1	Yes	HSD11B2	Yes	PDE5A	Yes
ADRA2C	Yes	CYP1A2	Yes	HSD17B10	Yes	PDE6H	Yes
ADRB1	Yes	CYP1B1	Yes	HSD17B3	Yes	PGR	Yes
ADRB2	Yes	CYP26A1	Yes	HSP90AA1	Yes	PKM	Yes

AGTR1	Yes	CYP2B6	Yes	HTR1A	Yes	PLG	Yes
AHCY	Yes	CYP2C19	Yes	HTR1B	Yes	PMP22	Yes
AHCYL1	No	CYP2C9	Yes	HTR1D	Yes	POLA1	Yes
AKR1C1	Yes	CYP2D6	Yes	HTR2A	Yes	POLB	Yes
AKR1C2	Yes	CYP3A4	Yes	HTR2B	Yes	PPARG	Yes
AKR1C3	Yes	CYP51A1	Yes	HTR2C	Yes	PPAT	Yes
ALDH1A1	Yes	DHFR	Yes	HTR3A	Yes	PPIA	Yes
ALOX15	Yes	DRD1	Yes	HTR4	Yes	PPP3CA	Yes
ALOX5	Yes	DRD2	Yes	HTR5A	Yes	PRSS1	Yes
ALOX5AP	Yes	DRD3	Yes	HTR6	Yes	PTGFR	Yes
AOC3	Yes	DRD4	Yes	HTR7	Yes	PTGS1	Yes
APEX1	Yes	EBP	Yes	HTT	Yes	PTGS2	Yes
AR	Yes	EGFR	Yes	IDO1	Yes	RAB9A	Yes
ASAH1	No	ERBB2	Yes	IFNA1	No	RECQL	Yes
ATP1A1	Yes	ERBB4	Yes	IMPDH1	Yes	RGS17	No
ATP4A	Yes	ESR1	Yes	IMPDH2	Yes	RIPK2	Yes
BCHE	Yes	ESR2	Yes	IRF3	No	RXRA	Yes
BHMT	Yes	F12	Yes	ITGAL	Yes	SCN1A	Yes
BLM	Yes	F2	Yes	KCNH2	Yes	SCNN1A	Yes
BMP2K	Yes	FABP3	No	KCNJ11	Yes	SERPINA6	No
C1S	Yes	FABP4	Yes	KCNJ8	Yes	SHBG	Yes
C4A	No	FDPS	Yes	KCNN4	Yes	SIGMAR1	Yes
C4B	No	FFAR1	Yes	KCNQ1	Yes	SIRT1	Yes
C5	No	FGF1	Yes	KDM4E	Yes	SLC12A1	Yes
CA1	Yes	FKBP1A	Yes	KDR	Yes	SLC12A3	Yes
CA12	Yes	FUT7	No	KIT	Yes	SLC22A12	Yes
CA14	Yes	GAA	Yes	KLF5	Yes	SLC22A6	Yes
CA2	Yes	GABBR1	Yes	KLRC3	No	SLC22A8	Yes
CA3	Yes	GABRA1	Yes	KLRK1	No	SLC29A1	Yes
CA4	Yes	GABRA2	Yes	KMT2A	Yes	SLC6A2	Yes
CA5B	Yes	GABRA3	Yes	L3MBTL1	Yes	SLC6A3	Yes

CA6	Yes	GABRA5	Yes	LMNA	Yes	SLC6A4	Yes
CA7	Yes	GAK	Yes	MAOA	Yes	SLC7A11	No
CA9	Yes	GALE	Yes	MAOB	Yes	SLCO1B1	Yes
CACNA1C	Yes	GFER	Yes	MAP2K1	Yes	SMN1	Yes
CACNA1G	Yes	GLO1	Yes	MAPK1	Yes	SQLE	Yes
CACNA1I	Yes	GMNN	Yes	MAPK10	Yes	TACR1	Yes
CBR1	Yes	GPR35	Yes	MAPK13	Yes	TACR2	Yes
CCL26	No	GPR55	Yes	MAPK14	Yes	TACR3	Yes
CCR4	Yes	GRIA4	Yes	MAPK9	Yes	TDP1	Yes
CD4	No	GRIK1	Yes	MAPT	Yes	THPO	Yes
CD46	No	GRIK2	Yes	MEN1	No	THRA	Yes
CD55	No	GRIK5	Yes	METAP2	Yes	THRB	Yes
CD8A	No	GRIN1	Yes	MGLL	Yes	TMEM97	No
CDK1	Yes	GRIN2A	Yes	MKNK2	Yes	TOP1	Yes
CDK2	Yes	GRK2	Yes	MT-ND4	Yes	TOP1MT	Yes
CDK4	Yes	GRM1	Yes	MTOR	Yes	TP53	Yes
CDK5	Yes	GRM4	Yes	NAPRT	No	TPO	Yes
CES1	Yes	GRM5	Yes	NFKB1	Yes	TRPA1	Yes
CHRM1	Yes	GSK3B	Yes	NISCH	Yes	TSHR	Yes
CHRM2	Yes	GSTP1	Yes	NPSR1	Yes	TUBB4B	Yes
CHRM3	Yes	HASPIN	Yes	NQO1	Yes	TYMS	Yes
CHRM4	Yes	HBB	Yes	NQO2	Yes	UMPS	Yes
CHRNA1	Yes	HDAC1	Yes	NR1I2	Yes	VKORC1	Yes
CHRNB2	Yes	HDAC11	Yes	NR3C1	Yes	WDR5	Yes

Supplementary Table 1: A table of proteins with corresponding druggability family information

3. Viral-host protein predictions

In silico identification of possible viral-host protein interactions is a challenging task. In recent years, several attempts have been made to find structural similarity from sequence (Zhou et al., 2014). We have implemented the sequence-based approach taking 11 MPXV proteins and BLASTing them (Altschul et al., 1990) towards human sequence SWISSPROT collection (Bairoch & Apweiler, 2000). Only human homologues with sequence identity >35% and only four of MPXV proteins (i.e., Q8V571 (p28), P04363 (TK), Q8V4S4 (B4R), Q8V4Y0 (E8L)) have been found having a sequence similarity with such a minimal

level. All four proteins, however, showed high similarity on relevant sequence lengths (in average ca. 200 residues). The list of the BLAST result is shown in **Supplementary Table 2**. The full BLAST result is available in https://github.com/Fraunhofer-ITMP/mpox-kg/tree/main/data/uniprot.

Mpox Protein (Uniprot)	Mpox Protein Names	Human Protein (Uniprot)	Human Protein Names	Percent Identity
Q8V571.1	p28	Q13064.1	MKRN3	47.143
Q8V571.1	p28	Q13434.1	MKRN4P	48.333
Q8V571.1	p28	Q9UHC7.3	MKRN1	38.889
Q8V571.1	p28	Q9H000.2	MKRN2	38.095
Q8V571.1	p28	O76064.1	RNF8	41.304
P04363.2	TK	P04183.2	TK1	67.836
Q8V4S4.1	B4R	Q8IYM2.2	SLFN12	34.983
Q8V4S4.1	B4R	Q6IEE8.4	SLFN12L	35.644
Q8V4Y0.1	E8L	P07451.3	CA3	37.281
Q8V4Y0.1	E8L	Q8N1Q1.1	CA13	37.555
Q8V4Y0.1	E8L	P35218.1	CA5A	35.484
Q8V4Y0.1	E8L	P00915.2	CA1	36.321
Q8V4Y0.1	E8L	P00918.2	CA2	36.889

Supplementary Table 2: A table summarizing BLAST results for MXPV proteins

Using this list of viral and human proteins, we created a subgraph of the KG. We found out that processes 'zinc ion binding' and 'carbonate dehydratase activity' were shared between human proteins CA1, CA2, CA3 and viral protein Q8V4Y0. Additionally, we also identifed 'metal ion binding' as a shared process between viral proteins p28 and TK (Supplementary Figure 3).

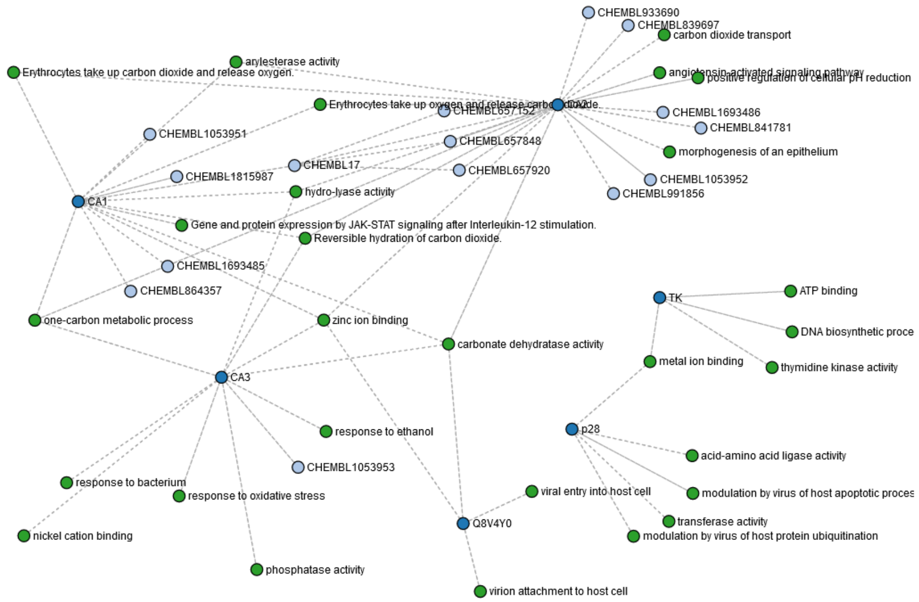

Supplementary Figure 3: A sub-graph depicting shared processes between human and viral proteins

4. Clinical Trial (Phase IV) drugs in Virus Diseases

The table below enlists 12 FDA-approved drugs that are used against Virus Diseases. Some drugs are prescribed against a specific virus disease such as Smallpox and Hepatitis C. The original file can be found at: https://github.com/Fraunhofer-ITMP/mpox-kg/tree/main/data/export/phase4drugs.csv.

Drug	max_phase	mesh_heading	Drug	max_phase	mesh_heading
CHEMBL116	4	Virus Diseases	CHEMBL1643	4	Fibrosis
CHEMBL1257073	4	Virus Diseases	CHEMBL1259059	4	Hepatitis C
CHEMBL1257073	4	Smallpox	CHEMBL1259059	4	Hepatitis C, Chronic
CHEMBL3137312	4	Virus Diseases	CHEMBL1259059	4	Virus Diseases
CHEMBL57	4	HIV Infections	CHEMBL61	4	Virus Diseases
CHEMBL57	4	HIV Infections	CHEMBL61	4	Condylomata Acuminata
CHEMBL57	4	Virus Diseases	CHEMBL61	4	Carcinoma, Squamous Cell
CHEMBL152	4	Virus Diseases	CHEMBL115	4	Virus Diseases
CHEMBL152	4	Cytomegalovirus Retinitis	CHEMBL163	4	Hepatitis C, Chronic
CHEMBL152	4	Primary Immunodeficiency Diseases	CHEMBL163	4	HIV Infections
CHEMBL853	4	Virus Diseases	CHEMBL163	4	HIV Infections
CHEMBL1643	4	Hepatitis C, Chronic	CHEMBL163	4	Hepatitis C
CHEMBL1643	4	Liver Cirrhosis	CHEMBL163	4	Virus Diseases
CHEMBL1643	4	Virus Diseases	CHEMBL584	4	Virus Diseases

Supplementary Table 3: A table listing FDA-approved/clinical trial (Phase IV) drugs in Virus Diseases

5. BLAST search for HIV reverse transcriptase (pol) and Mpox Protein OPG148 (UniProt: A0A7H0DNC0)

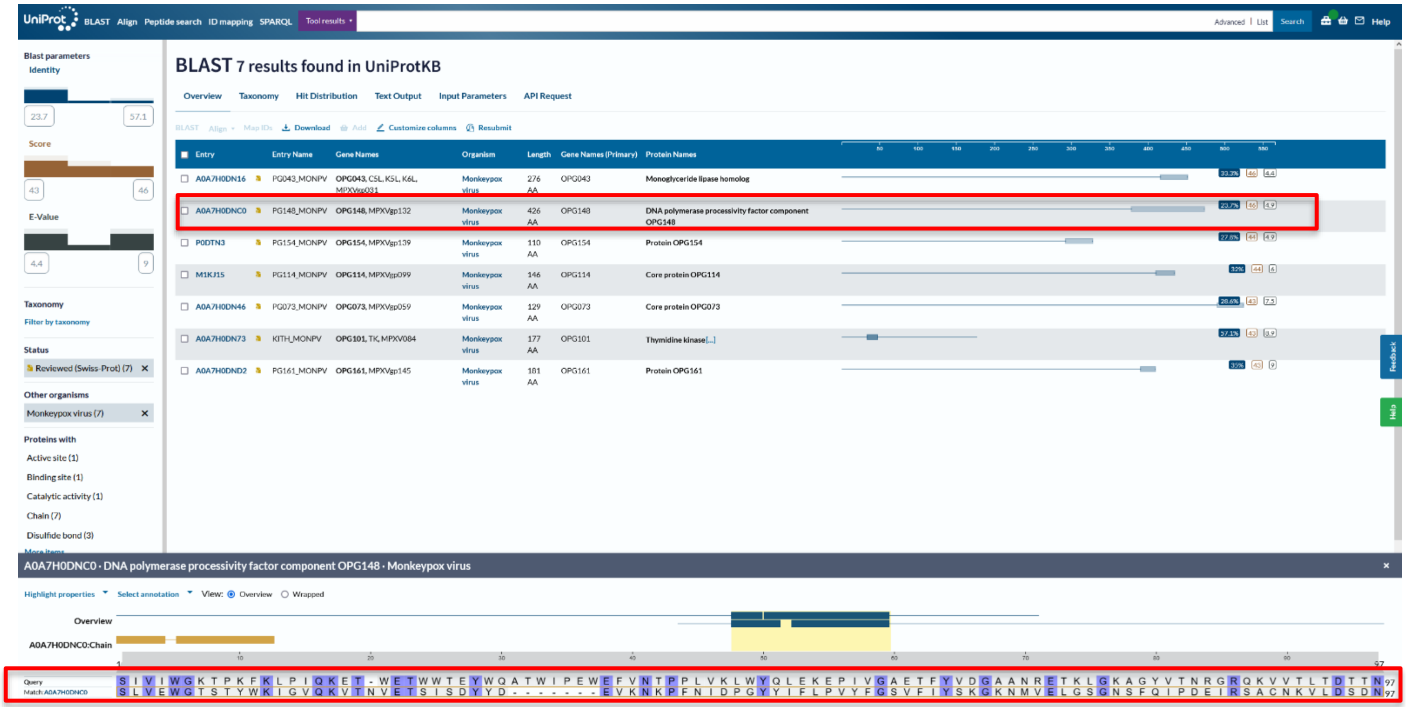

Supplementary Figure 4: BLAST search for HIV reverse transcriptase against Mpox proteins

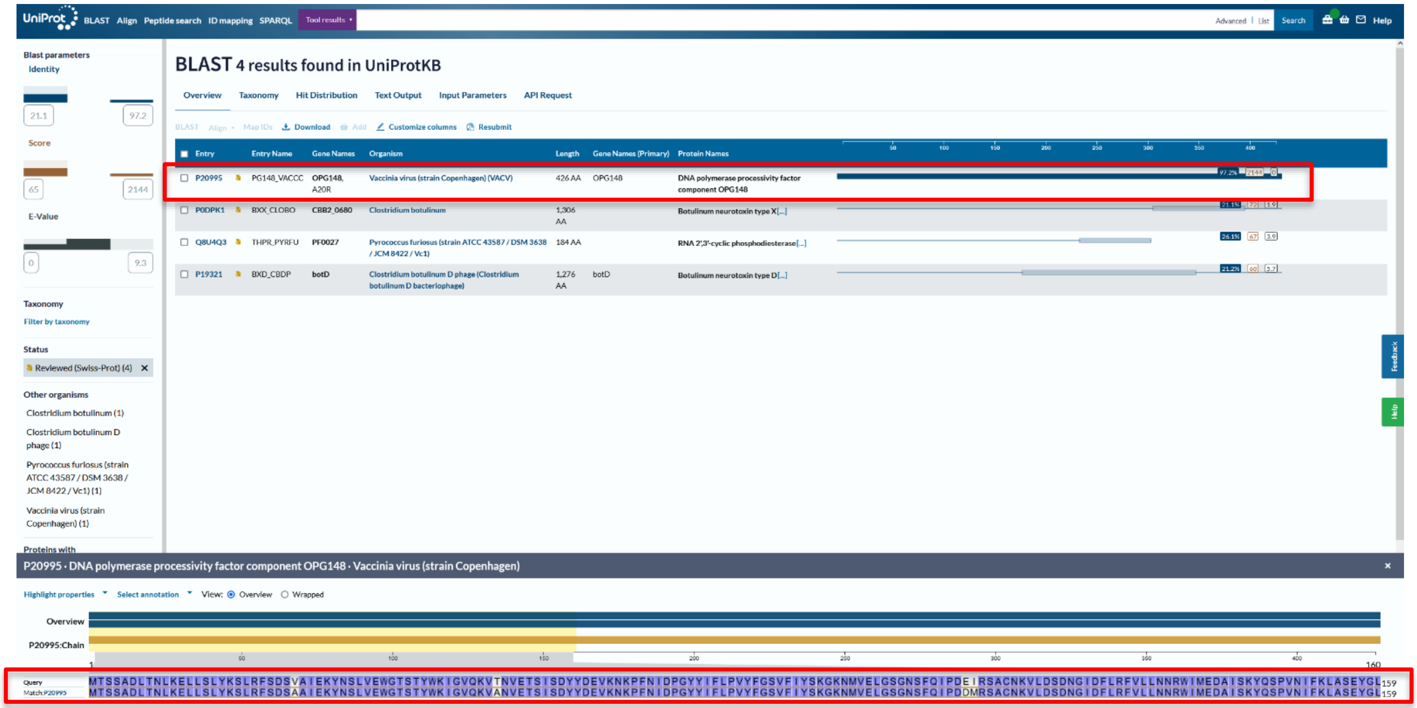

Supplementary Figure 5: BLAST search for Mpox OPG148 to identify its orthologs

6. Structural Similarity between Uracil- Nevirapine and Uracil- Zalcitabine

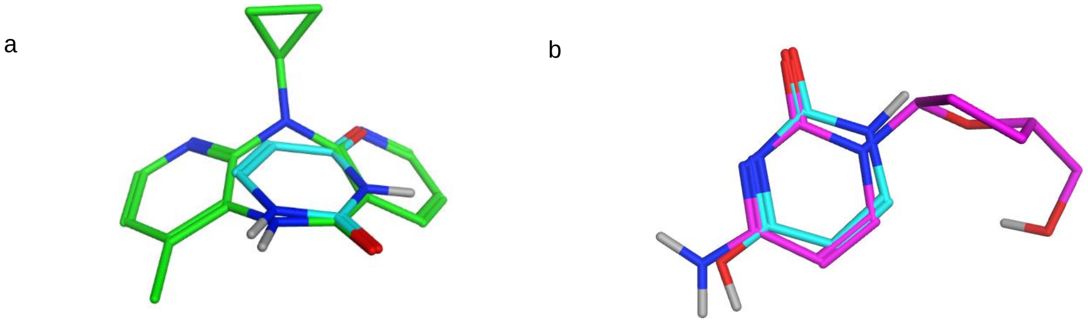

Supplementary Figure 6: Super-imposition of Uracil (color: cyan) against a) Nevirapine and b) Zalcitabine.

References

Altschul, S. F., Gish, W., Miller, W., Myers, E. W., & Lipman, D. J. (1990). Basic local alignment search tool. *Journal of Molecular Biology*, *215*(3), 403–410.

Bairoch, A., & Apweiler, R. (2000). The SWISS-PROT protein sequence database and its supplement TrEMBL in 2000. *Nucleic Acids Research*, *28*(1), 45–48.

Zhou, H., Gao, S., Nguyen, N. N., Fan, M., Jin, J., Liu, B., Zhao, L., Xiong, G., Tan, M., Li, S., & others. (2014). Stringent homology-based prediction of H. sapiens-M. tuberculosis H37Rv protein-protein interactions. *Biology Direct*, *9*(1), 1–30.
